# Clinical characteristics, visual acuity outcomes, and factors associated with loss of vision among patients with active ocular toxoplasmosis: A retrospective study in a Thai tertiary center

**DOI:** 10.1371/journal.pntd.0012232

**Published:** 2024-06-06

**Authors:** Wantanee Sittivarakul, Wanitcha Treerutpun, Usanee Tungsattayathitthan

**Affiliations:** Department of Ophthalmology, Faculty of Medicine, Prince of Songkla University, Hat Yai, Songkhla, Thailand; Statens Serum Institut, DENMARK

## Abstract

**Background:**

Ocular toxoplasmosis (OT) is the most common cause of infectious uveitis worldwide, including Thailand. This study describes the clinical presentation, visual acuity (VA) outcomes, and factors associated with VA loss in patients with active OT following antiparasitic treatment.

**Methodology/Principal findings:**

A retrospective chart review of patients with active OT treated with antiparasitic drugs between 2010 and 2020 was performed. Outcome measures included clinical characteristics, interval VA, and predictive factors associated with loss of VA ≤ 20/50 at 6 months post-treatment. Ninety-two patients (95 eyes) were enrolled. The median follow-up time was 10.9 months (IQR 4.9–31.8 months). The median age at presentation was 35.9 years, 51% were male, and 92.4% had unilateral OT. Eleven patients (12%) were immunocompromised (HIV infection, eight patients; receiving immunosuppressive agents, three patients). Patients mainly presented with primary retinitis without previous scar (62%), posterior pole lesion (56%), and lesion size of ≤ 2–disc area (75%). Immunocompromised patients showed a significantly larger size of retinitis than immunocompetent patients. Oral trimethoprim/sulfamethoxazole monotherapy was the primary short-term antiparasitic drug prescribed (85%). At the final visit, 21% of all affected eyes suffered VA ≤ 20/200. The cumulative incidence of recurrent OT at three years was 33.9% (95% CI, 19.7%–54.2%). Immunocompromised patients [adjusted odds ratio (aOR) 4.9, p = 0.041], macular lesion (aOR 5.4, p = 0.032), and initial VA ≤ 20/200 (aOR 9.1, p = 0.014) were predictive of having VA ≤ 20/50 at 6 months post-treatment.

**Conclusions:**

Ocular toxoplasmosis mainly presents as unilateral primary retinitis within the posterior pole. Severe VA loss was observed in one-fifth of eyes following treatment with lesion resolution. Immunocompromised patients, eyes with macular lesions, and poor initial VA were associated with poor VA outcomes.

## Author summary

Ocular toxoplasmosis (OT) is the most common cause of infectious uveitis worldwide, including Thailand. Vision loss is common despite receiving antiparasitic treatment because of the development of ocular complications. Little is known about the Thai population’s demographics, clinical presentations, and visual outcomes. We conducted a retrospective cohort study to examine the clinical presentation and visual acuity outcomes of this group of patients, including both immunocompetent and immunocompromised patients, at the largest tertiary center in southern Thailand. Patients with active OT in our cohort mainly presented with unilateral, focal retinitis without associated scar, and central lesions. Our findings support those of previous studies, in which immunocompromised patients exhibited significantly larger lesion sizes with poorer visual acuity recovery than immunocompetent patients. A significant proportion (21%) of all affected eyes showed severe vision loss following antiparasitic treatment, in which oral trimethoprim/sulfamethoxazole monotherapy was mainly used. Education on *Toxoplasma gondii* infection and transmission should be provided to communities to decrease the risk of infection and ocular toxoplasmosis disease burden.

## Introduction

*Toxoplasma gondii* is an obligate intracellular protozoan parasite belonging to the phylum Apicomplexa. It is classically described as one of the most successful parasites in the world, widely infecting humans, domesticated, and wild warm-blooded animals. [1] It is estimated that more than one-third of the global population harbors *T*. *gondii*. [2] The ocular presentation of this infection, ocular toxoplasmosis (OT), is known to be the most common cause of infectious uveitis worldwide, including in Thailand. A recent meta-analysis revealed that OT was responsible for 33% (95% CI, 24–42%) of cases of posterior uveitis. [3] Ocular involvement can occur without any significant evidence of systemic disease. Traditionally, OT has been attributed to the reactivation of congenital infections. However, collective evidence shows that most OT cases are acquired infections after birth [1,4], most often from the consumption of tissue cysts in undercooked meat, oocysts in unwashed garden produce, or contaminated water.

Diagnosis of OT mainly relies on clinical manifestations. The typical presentation of active OT is characterized by focal necrotizing retinitis with or without an adjacent hyperpigmented retinal scar due to a previous episode of OT, accompanied by vitritis and retinal vasculitis. [5] The presence of serum antibodies against *T*. *gondii* in the context of compatible ocular disease supports the diagnosis. More definitive tests like *T*. *gondii* DNA polymerase chain reaction (PCR) or intraocular antibody production for *T*. *gondii* are typically performed to confirm an infection in patients with atypical presentations. [6] Although retinal lesions are self-limiting in otherwise healthy individuals, the disease potentially causes significant visual acuity (VA) loss owing to the occurrence of vision-threatening complications such as macular scarring, vitreous opacities, epiretinal membrane (ERM), and choroidal neovascularization (CNV). In addition, OT reactivation, owing to the persistence of tissue cysts in retinal tissues, can increase the probability of VA loss. Previous studies showed that approximately 14%–32% of affected eyes suffered a VA of ≤ 6/60 after the resolution of acute episodes of OT. [7–9]

Previous studies have demonstrated variations in the epidemiology, clinical presentation, and visual outcomes of OT among different geographical regions. Differences in human genetic disposition and infecting parasite strains have been hypothesized to influence the prevalence and severity of ocular involvement. [5,10,11] In the south east Asia region, data related to the clinical presentation and visual outcomes of OT are limited. Additionally, information on OT in Thai patients is lacking. The present study aimed to describe the demographic characteristics, clinical presentations, treatment approaches, and VA outcomes of patients with active OT, including immunocompetent and immunocompromised individuals treated with antiparasitic drugs. In addition, the predictive factors associated with loss of VA to ≤ 20/50 at 6 months post-treatment were investigated.

## Methods

### Ethics statement

This study was approved by the Ethics Committee of the Faculty of Medicine, Prince of Songkla University (REC number 64-060-2-4). The Ethics Committee of the Faculty of Medicine, Prince of Songkla University, determined that written informed consent was not required for this study as the risks involved were minimal and a waiver would not adversely affect the rights and welfare of the study participants. Patient data were kept confidential and compliant with the tenets of the Declaration of Helsinki.

### Study population

A retrospective chart review of all patients diagnosed with active episodes of OT and managed at Songklanagarind Hospital, a major tertiary center in southern Thailand, between January 2010 and December 2020 was performed. The diagnosis of OT was based on the presence of focal or paucifocal active necrotizing retinitis with or without hyperpigmented and/or atrophic chorioretinal scars, combined with the presence of serological evidence of systemic infection with *T*. *gondii*, plus PCR of ocular fluid in selected cases. [12] In addition, a clinical response must be observed following standard antiparasitic treatment for OT. The inclusion criteria consisted of patients of all ages who were diagnosed with active OT and treated with antiparasitic therapy. Patients were also required to have a follow-up duration of six weeks or more. We excluded patients with quiescent chorioretinal scars consistent with OT but who never showed reactivation of retinitis throughout the follow-up period, those who were not examined during the active stage of the disease, those who were lost to follow-up before receiving antiparasitic treatment, and those with incomplete medical records. Patients were initially identified from the electronic uveitis database system created for the clinical record-keeping of all new consecutive patients seen at our uveitis clinic. The medical records of the identified patients were subsequently reviewed to include only those individuals who fulfilled the inclusion criteria.

### Data collection

The data collected included age, sex, laterality of ocular involvement, presenting symptoms, duration between the onset of ocular symptoms and presentation, duration of follow-up, systemic diseases affecting the patients’ immune status (HIV infection or other diseases that required chemotherapy or immunosuppressive agents), and serum *T*. *gondii* antibodies. The ophthalmological examination was performed using a slit lamp biomicroscope with a 90 D lens and/or an indirect ophthalmoscope with a 20 D lens in selected cases. The information collected included best-corrected VA (BCVA) using The Early Treatment of Diabetic Retinopathy Study (ETDRS) chart with logMAR conversion. The anatomic location, course of uveitis, grading of anterior chamber cells, and vitreous haze were categorized according to the Standardization of Uveitis Nomenclature (SUN) guidelines.[13] The location of active retinitis lesion was classified as posterior pole, which included the area within the vascular arcades or within 1500 μm of the margin of the optic disc; mid periphery, which is the area extending anteriorly from posterior pole to the equator; and periphery, which is the remaining retina that extends to the ora serrata. The following ocular complications were recorded at presentation and during follow-up: ocular hypertension (OHT, IOP >21 mmHg), posterior synechia, cataract (≥grade 1), macular scar, CNV, ERM, and retinal detachment (RD). Surgical procedures including cataract surgery and pars plana vitrectomy (PPV), were also recorded. OT recurrence was defined as new-onset active focal necrotizing retinitis either adjacent to or distant from preexisting retinochoroidal scars during the follow-up period.

The treatment data included the dose and duration of antiparasitic regimens and adjunctive systemic corticosteroids. Oral trimethoprim (TMP) /sulfamethoxazole (SMZ) (80 mg/400 mg) monotherapy, dosing two tablets twice daily, was the first-line regimen for all patients unless they were known to be allergic to sulfa derivatives, in which cases, alternative regimens such as oral clindamycin would be prescribed. Intravitreal clindamycin injection was also offered as primary or adjuvant treatment in patients who refused systemic treatment, were pregnant with recurrent OT, or showed poor clinical response to systemic antiparasitic agents.

### Outcome measurements

The main outcome measures were ophthalmologic characteristics at the presentation and pattern of mean BCVA of the affected eyes after receiving the treatment at 1, 4 weeks, 3 months ± 4 weeks, 6 months ± 4 weeks, 1 year ± 6 weeks, and 2 years ± 8 weeks. Secondary outcome measures included predictive factors associated with moderate visual loss (MVL), defined as a BCVA of ≤ 20/50 at 6 months post-treatment, and the cumulative incidence of recurrence of OT during follow-up. The 6-month post-treatment interval was chosen to evaluate the VA outcome, as we believe that this duration is suitable for VA recovery after complete lesion resolution. The eye-time at risk of retinitis recurrence was calculated beginning from the initial presentation of each affected eye and ending at the date when the first recurrence of the retinitis lesion in that eye was observed.

### Statistical analyses

All statistical analyses were performed using STATA version 14 (StataCorp LP, College Station, TX, USA). Descriptive statistics are reported as frequencies (%), means (standard deviation), or medians (interquartile range [IQR]), as appropriate. The longitudinal patterns of the mean BCVA during follow-up were analyzed using a mixed-effects random intercept linear regression model, in which the patient and eyes were considered random elements, and time was the fixed effect. Pearson’s chi-square test or Fisher’s exact test was used to analyze the relationships between categorical variables. The Mann–Whitney U and Kruskal–Wallis tests were used to analyze the relationship between continuous and categorical variables. Logistic regression models were used to identify potential factors associated with loss of BCVA to ≤ 20/50 at 6 months post-treatment. Variables showing some evidence of association with BCVA ≤ 20/50 at 6 months were included in the initial logistic regression model. The model was then refined by backward elimination of variables that did not contribute significantly to the fit of the model, guided by the change in the log likelihood of successive hierarchical models, retaining only those variables with a p-value <0.05. In the modeling process, missing values were accounted for by covariate adjustment. A Kaplan-Meier curve of the time to the development of the first recurrence of toxoplasmic retinitis after enrollment was constructed, and the log-rank test was used for survival comparisons.

## Results

### Demographics and general characteristics of the study population

**Fig 1:**
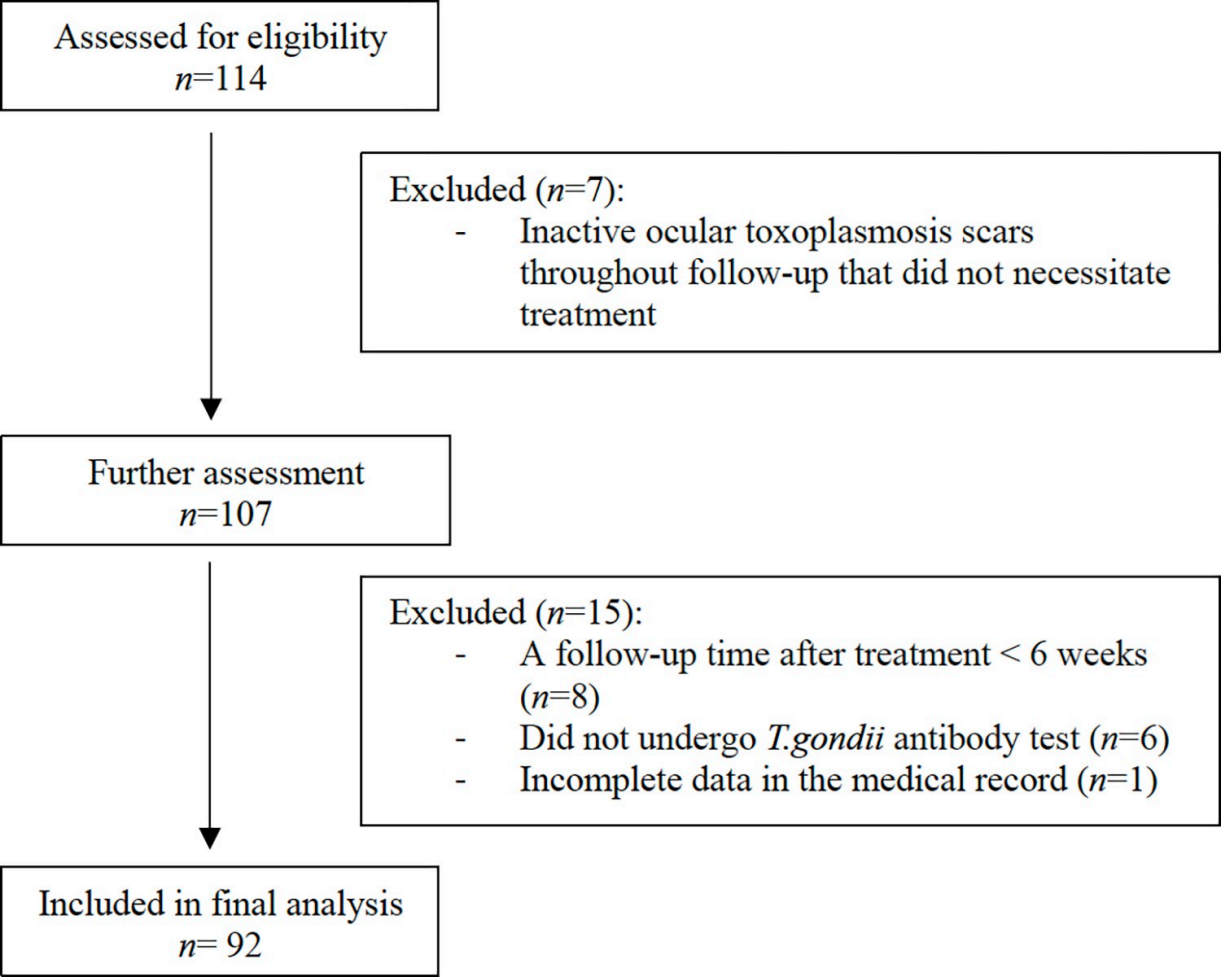
CONSORT flow diagram showing patient exclusion criteria for this retrospective study.

Initially, 114 patients diagnosed with OT during the study period were identified from the database. After reviewing their medical records, 22 patients were excluded from the study. Among them, seven patients had inactive OT scars throughout the follow-up period and did not necessitate treatment, eight patients had a follow-up time after treatment < 6 weeks, six patients did not undergo the *T*. *gondii* antibody test, and one patient had incomplete data in the medical records. Therefore, 92 patients (95 eyes) were included in this study (Fig 1).

The demographic characteristics of patients with active OT grouped according to immune status are presented in Table 1. The median age of patients was 35.9 years (IQR, 20.8–55.8 years). Males and females were nearly equal (51.1% and 48.9%, respectively). Eighty-five (92.4%) patients had unilateral OT. Eighty-one patients (88%) were immunocompetent. The remaining 11 (11.9%) patients were immunocompromised. Among them, eight had HIV infection [median CD4 counts of 124 cells/μL (IQR, 54–359 cells/μL)], two had leukemia and were receiving chemotherapy, and the other one had systemic lupus erythematosus and was receiving cyclophosphamide. The median follow-up time after presentation was 10.9 months (IQR 4.9–31.8 months). The demographics and characteristics of immunocompetent and immunocompromised patients at first presentation were not significantly different. All patients were positive for serum *T*. *gondii* IgG, whereas only three patients (3.3%) were positive for *T*. *gondii* IgM. The most common ocular symptoms were decreased vision (91.8%), redness (17.5%), and pain (13.4%).

**Table 1:** Demographics and characteristics of 92 patients with active ocular toxoplasmosis at first presentation.

		Immune status		
**Characteristics**	**Total** **n (%) or** **Median (IQR)** **(N = 92)**	**Immunocompetent** **n (%) or** **Median (IQR)** **(n = 81)**	**Immunocompromised** **n (%) or** **Median (IQR)** **(n = 11)**	**P value**
Age at presentation (years)	35.9 (20.8, 55.8)	32.0 (20.0, 56.2)	43.9 (31.9, 52.5)	0.122
Sex Male Female	47 (51.1) 45 (48.9)	41 (50.6) 40 (49.4)	6 (54.6) 5 (45.4)	0.807
Duration of ocular symptoms prior to presentation (days)	14.5 (7, 30)	14 (7, 30)	21 (14, 60)	0.088
Laterality Unilateral Bilateral Active lesions in both eyes at presentation Active lesions in one eye only at presentation	85 (92.4) 7 (7.6) 2 (2.2) 5 (5.4)	75 (92.6) 6 (7.4) 1 (1.2) 5 (6.2)	10 (90.9) 1 (9.1) 1 (9.1) 0 (0.0)	0.843
Duration of follow up (months)	10.9 (4.9, 31.8)	11.3 (4.9, 31.8)	8.0 (2.7, 20.3)	0.393
Antiparasitic regimens prescribed Oral TMP/SMZ monotherapy Oral TMP/SMZ and oral clindamycin Oral clindamycin monotherapy Oral TMP/SMZ and intravitreal clindamycin Intravitreal clindamycin alone	82 (89.1) 2 (2.2) 3 (3.3) 3 (3.3) 2 (2.2)	74 (91.3) 1 (1.2) 2 (2.5) 2 (2.5) 2 (2.5)	8 (72.7) 1 (9.1) 1 (9.1) 1 (9.1) 0 (0.0)	0.199

### Ophthalmologic characteristics

**Fig 2:**
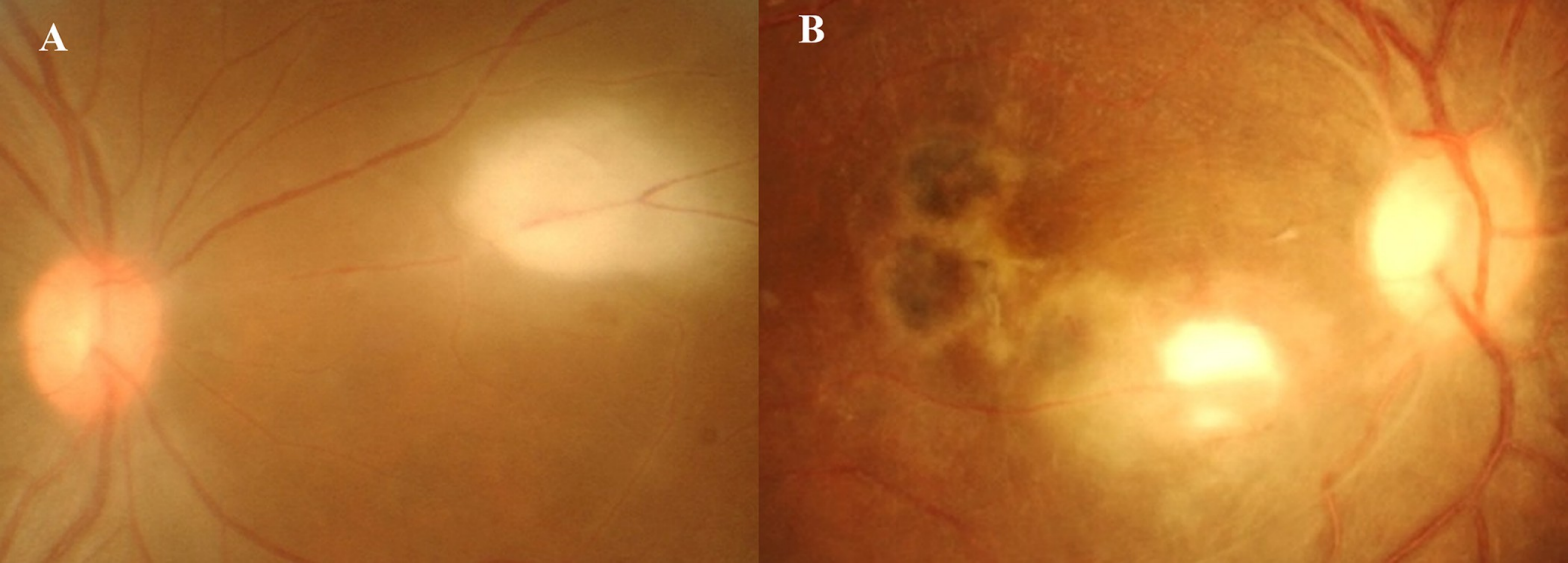
Fundus photographs of a case of ocular toxoplasmosis. (A) Area of focal active retinitis without an associated chorioretinal scar and (B) with an associated scar.

**Fig 3:**
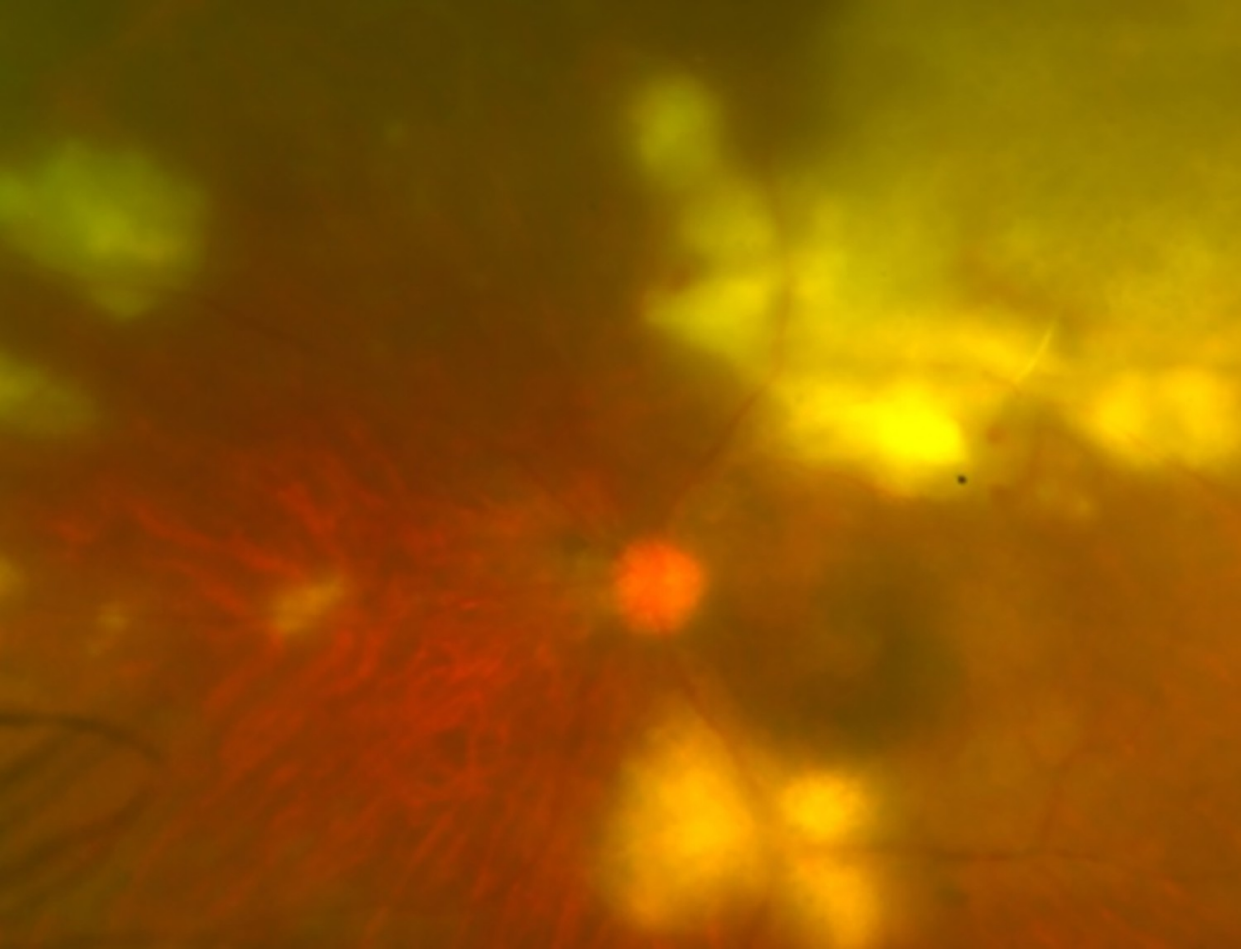
Fundus photograph of a patient with human immunodeficiency virus infection. Patient with human immunodeficiency virus infection with active ocular toxoplasmosis characterized by multifocal and extensive necrotizing retinitis.

The ophthalmological characteristics of the 95 eyes (92 patients) with active OT are presented in Table 2. Acute (81%), unilateral (92.4%), non-granulomatous (83.2%), and panuveitis (56.8%) were the main presentations of uveitis. Active retinitis lesions were mostly located in the posterior pole (55.8%), and macular involvement was observed in 44.2% of the eyes. Primary retinitis lesions without previously associated OT scars (Fig 2A) were observed in 62.1% of the eyes, whereas retinitis associated with chorioretinal scars was observed in 37.9% of the eyes (Fig 2B). Patients who presented with primary retinitis without scar were significantly older [median age = 41.9 years (IQR 23.4–57.8)] than those with scars at initial presentation [median age = 27 years (IQR 19.8–42.9)], p = 0.028. The mean presenting logMAR BCVA of all affected eyes was 0.86 ± 0.06 (Snellen equivalent of 20/145). Approximately 42% of affected eyes presented with BCVA ≤ 20/200. In comparisons between immunocompetent and immunocompromised patients, immunocompromised patients more frequently had a larger retinitis lesion size of >2-disc area (p = 0.008) (Fig 3) and an increased likelihood of worse final mean BCVA than immunocompetent patients (Snellen equivalent of 20/200 vs. 20/55, p < 0.001). There was no significant difference in the severity of cellular grading, number of active lesions per eye, or lesion location and proportion of eyes with and without previous scars between the two groups.

**Table 2:** Ophthalmological characteristics of 95 eyes (92 patients) with ocular toxoplasmosis at presentation according to immune status.

Characteristic	Total (N = 95)	Immune status		P value
		Immunocompetent (n = 83)	Immunocompromised (n = 12)	
Type of uveitis, n (%) Granulomatous Nongranulomatous	16 (16.8) 79 (83.2)	13 (15.7) 70 (84.3)	3 (25) 9 (75)	0.419
Course of uveitis, n (%) Acute Recurrent Chronic	77 (81) 9 (9.5) 9 (9.5)	67 (80.7) 7 (8.4) 9 (10.8)	10 (83.3) 2 (16.7) 0	0.357
Anatomic location, n (%) Posterior uveitis Panuveitis	41 (43.2) 54 (56.8)	37 (44.6) 46 (55.4)	4 (33.3) 8 (66.7)	0.545
Anterior chamber cells grade, n (%) 0 to trace 1+ to 2+ 3+ to 4+	39 (41.0) 32 (33.7) 24 (25.3)	34 (41.0) 29 (34.9) 20 (24.1)	5 (41.7) 3 (25.0) 4 (33.3)	0.718
Vitreous haze grade, n (%) 0 to trace 1+ to 2+ 3+ to 4+	37 (38.9) 49 (51.6) 9 (9.5)	34 (41.0) 43 (51.8) 6 (7.2)	3(25.0) 6 (50.0) 3 (25.0)	0.123
Median intraocular pressure, mmHg (IQR)	14 (11, 16)	14 (11, 16)	12 (10.5, 15)	0.268
Number of active lesions per eye, mean (SD)	1.2 (0.5)	1.2 (0.5)	1.3 (0.5)	0.723
Lesion location, n (%) Posterior pole Midperiphery Periphery	53 (55.8) 17 (17.9) 25 (26.3)	44 (53) 16 (19.3) 23 (27.7)	9 (75) 1 (8.3) 2 (16.7)	0.481
Lesion involved macular, n (%)	42 (44.2)	35 (42.2)	7 (58.3)	0.358
Lesion involved optic disc, n (%)	9 (9.5)	6 (7.2)	3 (25)	0.084
Lesion size (disc area), n (%) ≤ 2 > 2 Indeterminate (not photographed or dense vitritis)	70 (75.3) 23 (24.7) 2	65 (80.3) 16 (19.7) 2	5 (41.7) 7 (58.3) 0	0.008
Proximate/adjacent pigmented scars, n (%) Present Absent	36 (37.9) 59 (62.1)	33 (39.8) 50 (60.2)	3 (25.0) 9 (75.0)	0.526
Mean initial logMAR BCVA (SD) Median initial logMAR BCVA (IQR)	0.86 (0.06) 0.80 (0.3,1.6)	0.82 (0.07) 0.70 (0.3,1.3)	1.10 (0.24) 1.40 (0.2,1.9)	0.153 0.229
Initial BCVA, n (%) > 20/50 20/50–20/160 ≤ 20/200	29 (30.5) 26 (27.4) 40 (42.1)	25 (30.1) 25 (30.1) 33 (39.8)	4 (33.3) 1 (8.3) 7 (58.3)	0.260
Mean final logMAR BCVA (SD) Median final logMAR BCVA (IQR)	0.51 (0.06) 0.30 (0.1,0.9)	0.44 (0.05) 0.30 (0.1,0.8)	1.00 (0.25) 1.10 (0.1,1.7)	<0.001 0.075
Final BCVA, n (%) > 20/50 20/50–20/160 ≤ 20/200	51 (53.7) 24 (25.3) 20 (21)	47 (56.6) 22 (26.5) 14 (16.9)	4 (33.3) 2 (16.7) 6 (50)	0.046
Recurrence of retinitis during follow-up, n (%) No Yes	78 (82.1) 17 (17.9)	66 (79.5) 17 (20.5)	12 (100) 0	0.116

LogMAR: log of the minimum angle of resolution; BCVA: best-corrected visual acuity.

### Treatment

All the patients received short-term antiparasitic agents for active OT. Oral TMP/SMZ (400 mg/80 mg) was most frequently prescribed (87 [94.6%] patients). Among these, 82 patients received oral TMP/SMZ as a single agent, two received oral TMP/SMZ in combination with oral clindamycin, and the remaining three patients received oral TMP/SMZ combined with intravitreal clindamycin injections because of an incomplete response after oral TMP/SMZ alone. Three patients (3.3%) were prescribed oral clindamycin alone because of a known history of sulfa allergy. Intravitreal clindamycin was administered as the sole medication to two patients (2.2%) (one pregnant woman with recurrent OT, and one breastfeeding woman). The median duration of TMP/SMZ treatment was 8.9 weeks (IQR, 6.1–11.6). Adverse drug reactions following antiparasitic treatment were recorded in eight patients (9.3%). All the patients received oral TMP/SMZ therapy. Rashes were the only adverse drug reactions recorded. No serious events, such as Stevens-Johnson syndrome, were reported. Following the initiation of antiparasitic therapy, 75 patients (84.8%) received oral prednisolone to control the accompanying intraocular inflammation. The initial prednisolone dosage ranged 20–50 mg/day with tapering and discontinuation within 3 months. Following treatment, all but one eye demonstrated retinitis healing. One patient was lost to follow-up, when the lesion was incompletely healed.

A one-year course of intermittent low-dose oral TMP/SMZ (400 mg/80 mg) taken as one tablet three times per week as secondary prophylaxis treatment for recurrent OT was subsequently prescribed in 8/92 patients (8.7%). All patients were considered to be at a high risk of VA loss if their disease recurred, including those with macular lesions and those with a history of multiple recurrences. No adverse drug reactions were reported in patients who received secondary prophylactic therapy.

### Recurrences of ocular toxoplasmosis

Among the 95 eyes (92 patients) that were followed-up for over 154.5 eye-years (EYs), 17 eyes (17 patients) exhibited at least one new recurrent episode of toxoplasmic retinitis (incidence rate 0.11/EYs; 95% CI, 0.07–0.17). Among the 17 patients, 10 (58.8%) were male. The median age of patients was 32 years at first presentation (IQR, 22.4–46.3). Eight patients (47.1%) had retinitis lesions located in the posterior pole prior to reactivation. The majority of these patients (82.3%) received oral TMP/SMZ monotherapy as a short-term treatment for their previous active episodes.

**Fig 4:**
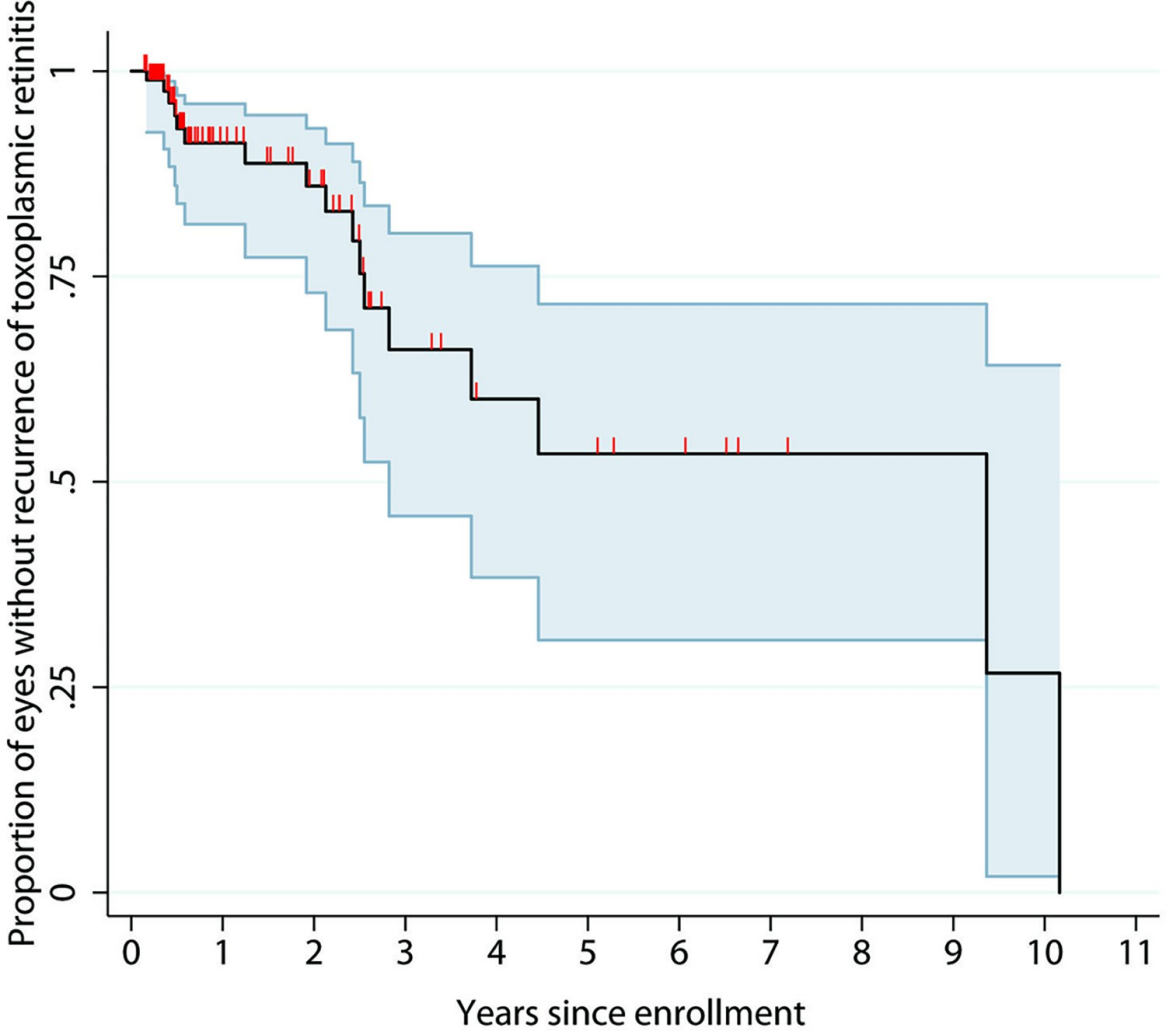
Kaplan-Meier curve of the overall cumulative probability. The graphic shows the overall cumulative probability of eyes free of ocular toxoplasmosis recurrence after an attack in 95 eyes. The shaded areas represent the 95% confidence intervals.

The Kaplan-Meier curve estimates of the overall cumulative incidence of first recurrent episodes of retinitis at 1, 2, and 3 years after enrollment were 8.8% (95% confidence interval [CI], 4.0%–18.6%), 14% (95% CI, 7.0%–27.0%), and 33.9% (95% CI, 19.7%–54.2%), respectively (Fig 4). Fourteen of the 17 patients (82%) recurred within three years of presentation. Univariate analyses (log-rank test) did not reveal any significant predictive factors associated with recurrent OT during the follow-up period. None of the patients who completed a 1-year course of secondary prophylactic therapy exhibited recurrence of OT over a median follow-up of 12 months (IQR 0.75–32.5 months) after prophylactic treatment discontinuation.

### Visual acuity outcome after treatment

Fifty-seven eyes (60%) demonstrated an improvement in BCVA of ≥ 2 lines, 31 eyes (32%) maintained BCVA within ±1 line, and seven eyes (7.4%) had a worsening in BCVA of > 1 line by the time of last follow-up. A final BCVA >20/50 and ≤20/200 was observed in 51 eyes (53.7%) and 20 eyes (21.1%), respectively. The primary cause of BCVA ≤ 20/200 was the most frequently due to macular scarring from OT lesions (17/20 eyes). In the remaining three eyes, VA loss was due to tractional RD (two eyes) and rhegmatogenous RD (one eye).

**Fig 5:**
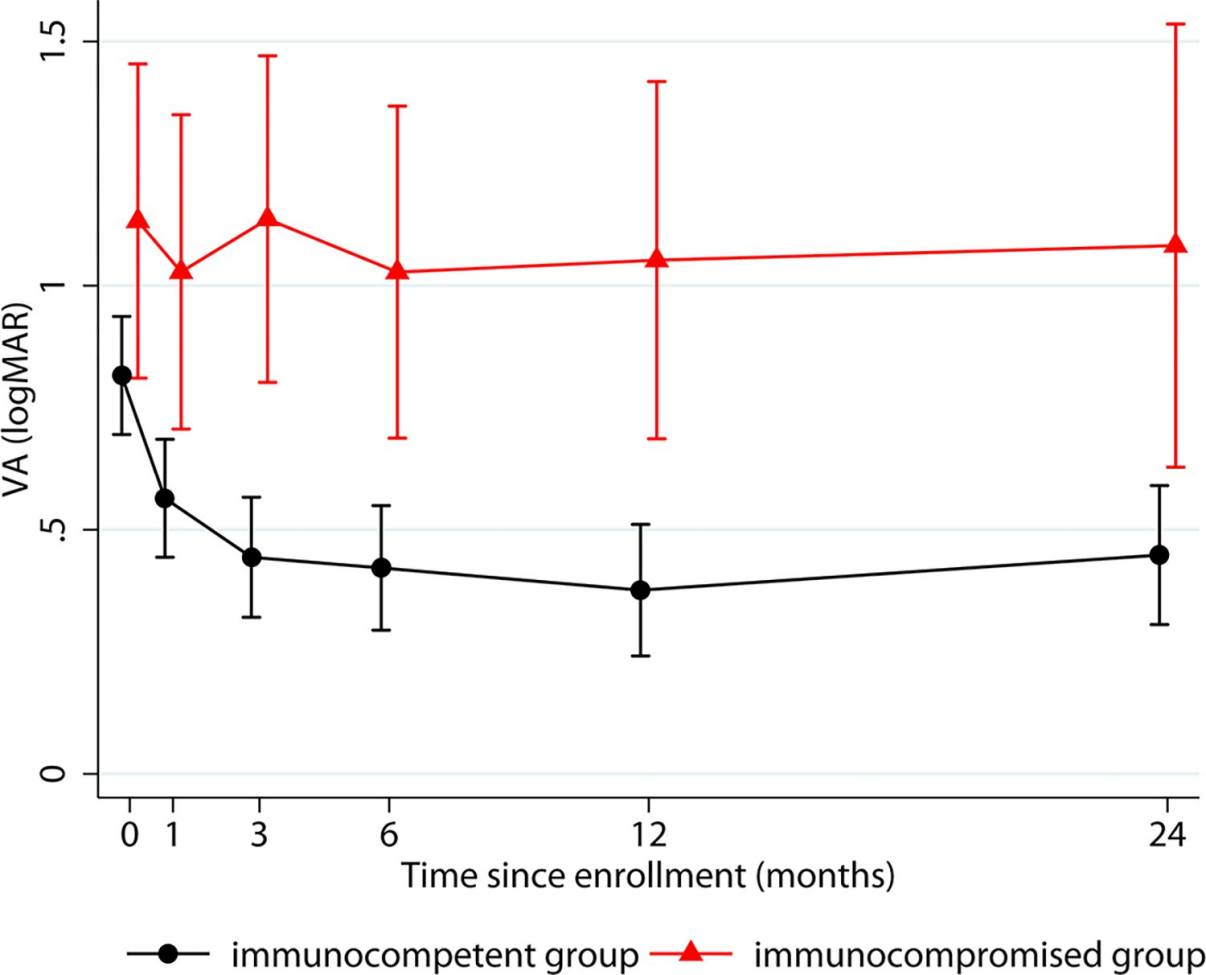
Estimates of mean logMAR best-corrected visual acuity over 2 years after presentation. The estimates were categorized according to the patients’ immune status using a linear mixed model. Vertical lines indicate the 95% confidence intervals.

Visual acuity observations derived from mixed-effects random-intercept linear regression over the first two years after presentation, categorized according to the patients’ immune status, are presented in Table 3. The modeled mean BCVA of the eyes of patients in the immunocompetent group was significantly better than that of the eyes of immunocompromised patients at every follow-up interval. The mean logMAR BCVA among eyes of immunocompetent patients significantly improved from 0.82 at presentation (95% confidence interval [CI], 0.70–0.94) (Snellen equivalent = 20/132) to 0.56 (95% CI, 0.44–0.68) (Snellen equivalent = 20/72) at 1 month, to 0.42 (95% CI, 0.29–0.55) (Snellen equivalent = 20/52) at 6 months, and stably maintained throughout the 2-year follow-up period. In contrast, the mean BCVA of the eyes of immunocompromised patients demonstrated non-significant improvement at every follow-up interval when compared with their baseline BCVA (Fig 5).

**Table 3:** Visual acuity outcomes after treatment in all eyes categorized according to immune status.

Outcome	Baseline	Month 1	Month 3	Month 6	Month 12	Month 24
Total eyes, n	95	95	81	63	44	31
No. of eye, n (immunocompetent group)	83	83	72	55	39	29
Mean logMAR BCVA (95% CI) of eyes in immunocompetent group ^a^	0.82 (0.70, 0.94)	0.56 (0.44, 0.68)	0.44 (0.32, 0.57)	0.42 (0.29, 0.55)	0.38 (0.24, 0.51)	0.45 (0.31, 0.59)
Snellen equivalent	20/132	20/72	20/55	20/52	20/47	20/56
P value^b^		<0.001	<0.001	<0.001	<0.001	<0.001
No. of eye, n (immunocompromised group)	12	12	9	8	5	2
Mean logMAR BCVA (95% CI) of eyes in immunocompromised group ^a^	1.13 (0.81, 1.45)	1.03 (0.71, 1.35)	1.14 (0.80, 1.47)	1.03 (0.69, 1.37)	1.05 (0.69, 1.42)	1.08 (0.63, 1.54)
Snellen equivalent	20/269	20/214	20/276	20/214	20/224	20/240
P value^b^		0.269	0.970	0.336	0.535	0.789
P value^c^	0.070	0.008	<0.001	0.001	0.001	0.009

LogMAR: log of the minimum angle of resolution; BCVA: best-corrected visual acuity.

^a^ Derived from the mixed-effect random intercept linear regression model.

^b^ Compared with baseline BCVA.

^C^ Comparison between the BCVA of the eyes in the immunocompetent and immunocompromised groups at each follow-up interval.

The univariate analyses of variables associated with loss of BCVA to ≤ 20/50 at 6 months are presented in Table 4. Of the 95 eyes, 63 (66.3%) were followed up at this time point and were included in the analyses. Eyes with retinitis located in the posterior pole (p < 0.001), eyes with lesion-involved macula (p < 0.001), and eyes with initial BCVA of ≤ 20/200 (p < 0.001) were more likely to have BCVA ≤ 20/50 at 6 months. A multivariate logistic regression analysis was performed (Table 5). Significant predictors associated with loss of BCVA to ≤ 20/50 at 6 months were: being an immunosuppressed patient [adjusted odds ratio (aOR) = 4.85, 95% CI, 1.07–22.07)], lesion involving the macula (aOR = 5.44, 95% CI, 1.16–25.45), and initial BCVA ≤ 20/200 (aOR = 9.12, 95% CI, 1.56–53.32)

**Table 4:** Comparison of eyes with and without best-corrected visual acuity loss to ≤ 20/50 at 6 months after treatment according to demographics and clinical characteristics.

Characteristics	BCVA at 6 months		P value	Crude Odds Ratio (95% CI)	P value
	VA > 20/50 (n = 34) no (%)	VA ≤ 20/50 (n = 29) no (%)			
Age at presentation (years) ≤36 >36	21 (61.8) 13 (38.2)	14 (48.3) 15 (51.7)	0.283	1 1.73 (0.62,4.80)	0.292
Duration of symptoms (days) ≤14 days >14 days	17 (50.0) 17 (50.0)	15 (51.7) 14 (48.3)	0.891	1 0.93 (0.34,2.54)	0.893
Sex Male Female	18 (52.9) 16 (47.1)	14 (48.3) 15 (51.7)	0.712	1 1.2 1(0.44,3.28)	0.715
Patients’ immune status Immunocompetent Immunocompromised	32 (94.1) 2 (5.88)	23 (79.3) 6 (20.7)	0.129	1 4.17 (0.93,18.76)	0.062
Uveitis course Acute Recurrent Chronic	29 (85.3) 1 (2.9) 4 (11.7)	21 (72.4) 4 (13.8) 4 (13.8)	0.259	1 5.52 (0.51,59.85) 1.38 (0.31,6.21)	0.160 0.674
Anterior chamber cells grade 0 to 1+ 2+ to 4+	19 (55.9) 15 (44.1)	17 (58.6) 12 (41.4)	0.827	1 0.89 (0.33,2.46)	0.828
Vitreous haze grade 0 to 1+ 2+ to 4+	25 (73.5) 9 (26.5)	23 (79.3) 6 (20.7)	0.591	1 0.72 (0.22,2.38)	0.595
Location of lesion Periphery Midperiphery Posterior pole	13 (38.2) 11 (32.4) 10 (29.4)	4 (13.8) 1 (3.4) 24 (82.8)	<0.001	1 0.30 (0.03,3.11) 7.80 (2.01,30.32)	0.310 0.003
Macular involvement No Yes	28 (82.4) 6 (17.6)	8 (27.6) 21 (72.4)	<0.001	1 12.25 (3.61,41.61)	<0.001
Lesion size (disc area) ≤ 2 >2	25 (75.8) 8 (24.2)	19 (67.9) 9 (32.1)	0.493	1 1.48 (0.46,4.71)	0.507
Presence of proximate/adjacent hyperpigmented scars No Yes	23 (67.7) 11 (32.3)	17 (58.6) 12 (41.4)	0.458	1 1.48 (0.51,4.24)	0.470
Initial BCVA ≤ 20/200 > 20/200	5 (14.7) 29 (85.3)	21 (72.4) 8 (27.6)	<0.001	1 0.07 (0.02,0.23)	<0.001
Recurrence of retinitis during follow up No Yes	26 (76.5) 8 (23.5)	20 (69.0) 9 (31.0)	0.504	1 1.46 (0.49,4.39)	0.498
Received prednisolone after antiparasitic therapy No Yes	3 (8.8) 31 (91.2)	7 (24.1) 22 (75.9)	0.097	1 0.30 (0.07, 1.30)	0.108

BCVA: best-corrected visual acuity.

**Table 5:** Multivariate logistic regression by robust estimation of predicting factors associated with best-corrected visual acuity loss to ≤ 20/50 at 6 months after treatment.

Characteristic	BCVA ≤20/50 at 6 months	
	Adjusted Odds Ratio (95% CI)	P value (Wald-test)
Duration of symptoms prior to presentation ≤ 14 days > 14 days	0.38 (0.06, 2.59)	0.327
Uveitis course Acute Recurrent Chronic	3.94 (0.49, 31.76) 6.63 (0.33, 133.63)	0.197 0.217
Host immune status Immunocompetent Immunocompromised	4.85 (1.07, 22.07)	0.041
Lesion involved macular No Yes	5.44 (1.16, 25.45)	0.032
Initial BCVA >20/200 ≤ 20/200	9.12 (1.56, 53.32)	0.014
Adjunctive prednisolone after antiparasitic therapy No Yes	0.26 (0.05,1.45)	0.124

BCVA: best-corrected visual acuity.

### Ocular complication and surgery

At least one ocular complication was noted in 61 eyes (64.2%) (Table 6). The most common complication was macular scarring (n = 24). Among the 24 eyes, macular scars were found at presentation in 11 eyes, and 13 eyes developed scars following a new episode of OT. OHT and ERM were observed less frequently. Two eyes and one eye developed tractional and rhegmatogenous RD, respectively, during follow-up.

**Table 6:** Ocular complications at presentation and during follow-up.

Complications	n (%)
Macular scar	24 (24.7)
Ocular hypertension (IOP >21 mmHg)	20 (20.6)
Epiretinal membrane	12 (12.6)
Cataract	6 (6.2)
Posterior synechia	5 (4.9)
Tractional retinal detachment	2 (2.1)
Rhegmatogenous retinal detachment	1 (1.1)

Six patients (six eyes) underwent ocular surgery. Among them, two underwent phacoemulsification (PE) with intraocular lens implantation, two underwent PPV (one eye for RRD repair and one eye for clearing vitreous opacity), and two underwent combined PE and PPV for cataract removal and clearing vitreous opacity. Two patients with TRD did not undergo PPV due to loss to follow-up (one eye) or severe inoperable TRD (one eye).

## Discussion

This study reports the clinical presentations, treatment approaches, and VA outcomes of both immunocompetent and immunocompromised patients with active OT. Most patients were young and presented with unilateral panuveitis. Roughly two-fifths of the eyes presented with BCVA ≤ 20/200, and half of the active retinitis lesions were located in the posterior pole. Oral TMP/SMZ was our cohort’s most commonly prescribed antiparasitic therapy for acute ocular manifestations. Following treatment, approximately 17% and 50% of immunocompetent and immunocompromised patients, respectively, had permanent severe VA loss in at least one eye at the final follow-up. The visual loss was mostly due to macular scarring. Immunocompromised patients tended to have larger retinitis lesions and showed poorer VA recovery than immunocompetent patients. During follow-up, approximately one-third of the eyes developed recurrent episodes of OT by three years after presentation.

Small community-based studies from some provinces in Thailand reported that the seroprevalence of toxoplasmosis, either among blood donors or pregnant women, varied from 2.5% to 28.3%.[14–16] In regards to ocular involvement, to date, no data are available regarding the epidemiology of OT in Thailand. However, OT is the most common cause of posterior/panuveitis in both adult and pediatric patients at our center, accounting for 7% and 12% of all patients with uveitis, respectively. Notably, the prevalence of OT at our uveitis center, located in southern Thailand, was much higher than that reported from central metropolitan centers.[17] This higher prevalence may be explained by sociocultural factors, eating/drinking habits, and occupations that were more contaminated with soil and water in people in the south compared to people in the central/metropolitan region of Thailand, resulting in a higher prevalence of OT.

The demographics of our patients were consistent with those of most previous reports from several regions worldwide. Most patients were young adults (median age, 36 years) without a sex preference. [7,9,18,19] Notably, the proportion of patients with primary lesions without scarring observed in our study was higher than that of patients with recurrent disease associated with scarring (62% vs. 38%). This finding was consistent with a study from a tertiary center in northern Thailand, where primary lesions were observed in 72% of all patients. [20] However, this observation contradicts that in several publications in which patients with recurrent disease associated with scarring were more frequently described. [6–9,21] We speculated that this finding is due to the differences in patient inclusion criteria between the studies. Most studies included both active and chronically inactive OT cases, unlike this study, in which only active cases were included. Hence, the previous studies may have included a larger number of patients with associated scars than those with primary retinitis lesions without scarring. In addition, immunocompromised patients were also included in our cohort. These patients are known to exhibit primary retinitis without scarring more frequently than immunocompetent individuals. The relatively high proportion of patients with primary lesions might also reflect a referral bias, given that focal retinitis without scarring contains a greater variety of uveitis differential diagnoses than those with associated scars, which is a classic presentation of OT. Thus, ophthalmologists at the community hospital were confident in diagnosing and managing classic OT cases without requiring referral.

Ocular toxoplasmosis in immunocompromised patients tends to present with a more severe course with extensive, multifocal lesions and no associated scarring compared to that in immunocompetent patients. In addition, a higher rate of systemic *T*. *gondii* dissemination has been described. [22,23] The present study supports these findings as we observed that immunocompromised patients showed a significantly larger area of retinitis than those with an intact immune status. In addition, 9/11 patients (81.8%) with HIV infection had primary lesions without associated scars. Diagnosing these cases was also more challenging given their extensive nature, mimicking other infectious posterior uveitis. PCR of intraocular fluid for *T*. *gondii* was carried out in 4/11 immunocompromised cases, all of which were positive. Computed tomography (CT) brain was also performed in 4/8 HIV-infected cases, two of which showed intracranial lesions compatible with cerebral toxoplasmosis.

The VA results during follow-up, demonstrated by mixed-effects random-intersect linear regression, suggest that the VA outcomes of immunocompetent patients with OT are relatively favorable. The patients’ VA improved soon after the initiation of treatment and was well maintained after 3 months. In contrast, immunocompromised patients exhibited significantly poorer VA levels throughout the follow-up period than immunocompetent patients. In addition, the VA following treatment appeared to have no changes compared to the baseline VA. This poor visual outcome is likely explained by a more extensive retinochoroidal lesion and the development of RD in some patients.

There is still significant uncertainty regarding the optimal treatment for OT. To date, there is no consensus on the best antiparasitic drug regimen for acute OT treatment. Given that OT is a self-limiting disease, and no drug has been shown to be truly curative owing to its ineffectiveness against dormant tissue cysts [24], some clinicians opt not to prescribe antiparasitic drugs to immunocompetent patients with small peripheral lesions owing to concerns about antiparasitic drug-related toxicity. Others treat all patients with active lesions aiming to control parasite multiplication, limit the size of the chorioretinal scar, and perhaps reduce recurrence. [25,26] However, recent surveys have demonstrated a trend shift towards a more widespread use of antiparasitic drugs among uveitis specialists over the past two decades, since the newer data have described a better tolerated and potentially more effective treatment. [6,27] The most recent survey conducted by the International Ocular Toxoplasmosis Study Group demonstrated that a majority of respondents (66%) treated all non-pregnant patients with active OT independent of the severity and location of the disease. This survey revealed eight different systemic antiparasitic drugs used for active OT. Oral TMP-SMZ was the most preferred treatment used by the respondents (67%), followed by clindamycin (27%). [6] These findings were consistent with our practice, in which we typically treated all patients with active lesions with antiparasitic agents regardless of lesion size or location. Moreover, oral TMP-SMZ was our first-line treatment because oral TMP-SMZ is inexpensive and widely available in Thailand. Our study showed that oral TMP-SMZ was well-tolerated. Approximately 9% of the patients reported adverse effects (rashes) that resolved after drug discontinuation. However, the incidence of rash was slightly higher than that reported in Brazil, where rash was observed in 4% of patients treated with antiparasitic drugs. [28]

The factors influencing OT recurrence remain uncertain. The age of the patient, duration since the first episode of OT, and the serotype of the parasite have been reported as factors associated with recurrence. [10,26,29] However, none of these clinical parameters was identified as an independent predictive factor for recurrent OT in the present study. A meta-analysis for the prevalence of recurrent OT demonstrated a global prevalence of 49% (95% CI, 40%–58%), in which the highest prevalence was reported in South America at 56%, followed by Central America at 51%. [30] Another study from Brazil reported the incidence of recurrent OT at 0.17/EY among eyes presenting with active episodes of OT. [9] Our recurrence rate appeared to be lower than in those studies. The overall proportion of patients with varying follow-up times who experienced recurrence was 18.5%, whereas the incidence of OT recurrence was 0.11/EYs when accounting for differential follow-up. However, there were inconsistencies in the inclusion criteria between the studies. Some studies have included only active cases, whereas others have included both active and chronically inactive cases. In addition, the treatment approaches and patient follow-up durations varied. These factors may have affected the interpretation of recurrent OT outcomes, making outcome comparisons difficult. In our study, no OT recurrence was observed in immunocompromised patients. However, the larger proportion of immunocompromised patients lost to follow-up over time compared with that of immunocompetent patients may have contributed to this outcome. Although none of the patients who had received secondary prophylaxis treatment with low-dose TMP-SMZ in our cohort exhibited recurrent OT during follow-up, the number of cases in which the prophylactic regimen was prescribed (8/92 cases) was too small to draw a firm conclusion on the efficacy of such a regimen. Further studies with a larger number of patients and longer specified follow-up intervals are required.

The present study demonstrated that approximately half of patients suffered BCVA ≤ 20/50 in at least one eye at 6 months after treatment. The predictive factors associated with BCVA ≤ 20/50 included being immunocompromised status, presenting BCVA ≤ 20/200, and having macular lesions. These associations were mostly consistent with previous studies in which they observed that final VA loss was linked to macular lesions, atypical presenting features, and the presence of any ocular complication.[8,9] Our findings showed no significant differences in VA outcomes at 6 months post-treatment between those who received and did not receive adjunctive oral prednisolone following antiparasitic therapy. However, this result should be interpreted with caution owing to the non-standardized doses and duration of prednisolone prescribed in our patients. Patients with central lesions or severe vitreous inflammation or who were immunocompetent were more likely to be prescribed prednisolone, which could have introduced indication bias. Bosch-Driessen et al. from the Netherlands also observed that patients with congenital toxoplasmosis had an increased likelihood of achieving poorer VA outcomes. [7] Our study did not analyze VA outcomes according to the mode of transmission, as there was no serological method to distinguish between these two groups reliably. Severe VA loss at the final follow-up was identified in 21% of patients in our cohort. This proportion was in the same range as that reported in studies conducted in Brazil, India, and Singapore [8,9,19], but higher than those conducted in Turkey and Iran, which reported a severe visual loss rate of approximately 10%. [21,31] The lower rates observed in these two latter studies could be attributed to the fact that they did not include patients with atypical disease and/or immunocompromised status.

Education regarding *T*. *gondii* infection and transmission should be provided to communities to reduce the risk of infection and decrease the ocular toxoplasmosis disease burden. Intervention measures could involve modifying eating habits, such as washing fruits and vegetables that are consumed raw, proper heating of all meat, avoiding raw meat products, and cleaning cooking utensils after contact with raw meat and seafood. [32]

The present study had several limitations. The retrospective nature of this study resulted in incomplete data and varied patient follow-up durations. Patients lost to follow-up may have led to an underestimation of the incidence of OT recurrence. In addition, differential loss to follow-up may have affected the longitudinal VA outcome, as patients with worse visual outcomes or with immunocompromised conditions were more likely to continue with follow-up than those with good visual outcomes and no systemic comorbidities. Our results demonstrate that most immunocompetent patients achieved significant VA improvement following TMP/SMZ monotherapy treatment as the main antiparasitic regimen. We acknowledge that the VA outcome alone may not be interpreted as a surrogate for therapeutic success. The visual outcome of patients with OT largely depends on the location of the lesion and whether they develop ocular complications independent of antiparasitic treatment. Other limitations also apply, including the possibility of unrecognized factors related to VA outcomes and/or recurring OT, such as parasite strain, host biological factors, or multimodal imaging findings that were not included due to the unavailability of data, and uncertain generalizability of the findings to populations in other regions of Thailand, in which the genotype of the infecting parasite and environmental factors may differ. Referral bias is also concerning, as the study was conducted at a single tertiary center; hence, there is a possibility of recruiting patients with a more severe clinical course. Nevertheless, our hospital is the only uveitis referral center in southern Thailand that serves patients with all types of uveitis. Therefore, we believe that our data represent the clinical information of patients with OT in this region and are not limited to tertiary settings. The study results could contribute to the data on OT from Southeast Asia, where infectious uveitis remains a significant etiology.

In summary, the present study demonstrated that patients with active OT in our cohort mainly presented with unilateral, primary focal retinitis without an associated scar, and central lesion location. Immunocompromised patients exhibited significantly larger lesion sizes and poorer VA recovery than immunocompetent patients following treatment. Oral TMP/SMZ, the main prescribed antiparasitic regimen, showed good tolerability and resolved retinitis lesions. Following treatment, approximately 17% and 50% of immunocompetent and immunocompromised patients, respectively, had permanent severe VA loss in at least one eye at the final follow-up. Retinitis recurrence was relatively common and most often occurred within three years of the first presentation. The genotypes of infecting parasites and ocular fluid biomarkers should be studied further to provide a better understanding of the pathophysiology and associated visual outcomes.

## Supporting information

S1 STROBE Checklist STROBE Checklist. (PDF)

S1 File Raw data. (XLSX)

## References

[1] Holland GN. Ocular toxoplasmosis: a global reassessment. Part I: epidemiology and course of disease. Am J Ophthalmol. 2003;136:973–988. doi: 10.1016/j.ajo.2003.09.040 14644206

[2] Robert-Gangneux F, Darde ML. Epidemiology of and diagnostic strategies for toxoplasmosis. Clin Microbiol Rev. 2012;25:264–296. doi: 10.1128/CMR.05013-11 22491772 PMC3346298

[3] Karami M, Gorgani-Firouzjaee T, Rostami-Mansour S, Shirafkan H. Prevalence of ocular toxoplasmosis in the general population and uveitis patients: a systematic review and meta-analysis. Ocul Immunol Inflamm. 2023:1–14. doi: 10.1080/09273948.2023.2190801 37043543

[4] Gilbert RE, Stanford MR. Is ocular toxoplasmosis caused by prenatal or postnatal infection? Br J Ophthalmol. 2000;84:224–226. doi: 10.1136/bjo.84.2.224 10655202 PMC1723371

[5] Holland GN. Ocular toxoplasmosis: a global reassessment. Part II: disease manifestations and management. Am J Ophthalmol. 2004;137:1–17. 14700638

[6] Yogeswaran K, Furtado JM, Bodaghi B, Matthews JM, Smith JR. Current practice in the management of ocular toxoplasmosis. Br J Ophthalmol. 2023;107:973–979. doi: 10.1136/bjophthalmol-2022-321091 35197262

[7] Bosch-Driessen LE, Berendschot TT, Ongkosuwito JV, Rothova A. Ocular toxoplasmosis: clinical features and prognosis of 154 patients. Ophthalmology. 2002;109:869–878. doi: 10.1016/s0161-6420(02)00990-9 11986090

[8] Aleixo AL, Curi AL, Benchimol EI, Amendoeira MR. Toxoplasmic retinochoroiditis: clinical characteristics and visual outcome in a prospective study. PLoS Negl Trop Dis. 2016;10:e0004685. doi: 10.1371/journal.pntd.0004685 27136081 PMC4852945

[9] Arruda S, Vieira BR, Garcia DM, Araújo M, Simões M, Moreto R, et al. Clinical manifestations and visual outcomes associated with ocular toxoplasmosis in a Brazilian population. Sci Rep. 2021;11:3137. doi: 10.1038/s41598-021-82830-z 33542439 PMC7862362

[10] Shobab L, Pleyer U, Johnsen J, Metzner S, James ER, Torun N, et al. Toxoplasma serotype is associated with development of ocular toxoplasmosis. J Infect Dis. 2013;208:1520–1528. doi: 10.1093/infdis/jit313 23878321 PMC3789564

[11] Smith JR, Ashander LM, Arruda SL, Cordeiro CA, Lie S, Rochet E, et al. Pathogenesis of ocular toxoplasmosis. Prog Retin Eye Res. 2021;81:100882. doi: 10.1016/j.preteyeres.2020.100882 32717377

[12] Classification Criteria for Toxoplasmic Retinitis. Am J Ophthalmol. 2021;228:134–141. doi: 10.1016/j.ajo.2021.03.042 33845002 PMC8594742

[13] Jabs DA, Nussenblatt RB, Rosenbaum JT. Standardization of uveitis nomenclature for reporting clinical data. Results of the First International Workshop. Am J Ophthalmol. 2005;140:509–516. doi: 10.1016/j.ajo.2005.03.057 16196117 PMC8935739

[14] Nissapatorn V, Suwanrath C, Sawangjaroen N, Ling LY, Chandeying V. Toxoplasmosis-serological evidence and associated risk factors among pregnant women in southern Thailand. Am J Trop Med Hyg. 2011;85:243–247. doi: 10.4269/ajtmh.2011.10-0633 21813842 PMC3144820

[15] Pinlaor S, Ieamviteevanich K, Pinlaor P, Maleewong W, Pipitgool V. Seroprevalence of specific total immunoglobulin (Ig), IgG and IgM antibodies to Toxoplasma gondii in blood donors from Loei Province, Northeast Thailand. Southeast Asian J Trop Med Public Health. 2000;31:123–127. 11023078

[16] Maleewong W, Lulitanond V, Pipitgool V, Auwijitaroon Y, Kuttsarejariya S, Morakote N. Prevalence of toxoplasma antibodies in blood donors and pregnant women in Khon Kaen Province. J Med Assoc Thai. 1989;72:256–259. 2788689

[17] Silpa-Archa S, Noonpradej S, Amphornphruet A. Pattern of uveitis in a referral ophthalmology center in the central district of Thailand. Ocul Immunol Inflamm. 2015;23:320–328. doi: 10.3109/09273948.2014.943773 25111619

[18] de-la-Torre A, López-Castillo CA, Gómez-Marín JE. Incidence and clinical characteristics in a Colombian cohort of ocular toxoplasmosis. Eye (Lond). 2009;23:1090–1093. doi: 10.1038/eye.2008.219 18617902

[19] Huang PK, Jianping C, Vasconcelos-Santos DV, Arruda JS, Dutta Majumder P, Anthony E, et al. Ocular toxoplasmosis in tropical areas: analysis and outcome of 190 patients from a multicenter collaborative study. Ocul Immunol Inflamm. 2018;26:1289–1296. doi: 10.1080/09273948.2017.1367407 29020481

[20] Pathanapitoon K, Kunavisarut P, Rothova A. Focal chorioretinitis in Thailand. Retina. 2014;34:587–591. doi: 10.1097/IAE.0b013e3182a1fac9 23928678

[21] Tugal-Tutkun I, Corum I, Otük B, Urgancioglu M. Active ocular toxoplasmosis in Turkish patients: a report on 109 cases. Int Ophthalmol. 2005;26:221–228. doi: 10.1007/s10792-007-9047-8 17318320

[22] Holland GN. Ocular toxoplasmosis in the immunocompromised host. Int Ophthalmol. 1989;13:399–402. doi: 10.1007/BF02306488 2697706

[23] de-la-Torre A, Gómez-Marín J. Disease of the year 2019: ocular toxoplasmosis in HIV-infected patients. Ocul Immunol Inflamm. 2020;28:1031–1039. doi: 10.1080/09273948.2020.1735450 32162993

[24] Feliciano-Alfonso JE, Muñoz-Ortiz J, Marín-Noriega MA, Vargas-Villanueva A, Triviño-Blanco L, Carvajal-Saiz N, et al. Safety and efficacy of different antibiotic regimens in patients with ocular toxoplasmosis: systematic review and meta-analysis. Syst Rev. 2021;10:206. doi: 10.1186/s13643-021-01758-7 34275483 PMC8287816

[25] Pradhan E, Bhandari S, Gilbert RE, Stanford M. Antibiotics versus no treatment for toxoplasma retinochoroiditis. Cochrane Database Syst Rev. 2016;2016:CD002218. doi: 10.1002/14651858.CD002218.pub2 27198629 PMC7100541

[26] Reich M, Ruppenstein M, Becker MD, Mackensen F. Time patterns of recurrences and factors predisposing for a higher risk of recurrence of ocular toxoplasmosis. Retina. 2015;35:809–819. doi: 10.1097/IAE.0000000000000361 25299969

[27] Morais FB, Arantes T, Muccioli C. Current practices in ocular toxoplasmosis: a survey of Brazilian uveitis specialists. Ocul Immunol Inflamm. 2018;26:317–323. doi: 10.1080/09273948.2016.1215471 27598330

[28] Casoy J, Nascimento H, Silva LMP, Fernández-Zamora Y, Muccioli C, et al. Effectiveness of treatments for ocular toxoplasmosis. Ocul Immunol Inflamm. 2020;28:249–255. doi: 10.1080/09273948.2019.1569242 30806556

[29] Holland GN, Crespi CM, ten Dam-van Loon N, Charonis AC, Yu F, Bosch-Driessen LH, et al. Analysis of recurrence patterns associated with toxoplasmic retinochoroiditis. Am J Ophthalmol. 2008;145:1007–1013. doi: 10.1016/j.ajo.2008.01.023 18343351

[30] Cifuentes-González C, Rojas-Carabali W, Pérez ÁO, Carvalho É, Valenzuela F, Miguel-Escuder L, et al. Risk factors for recurrences and visual impairment in patients with ocular toxoplasmosis: A systematic review and meta-analysis. PLoS One. 2023;18:e0283845. doi: 10.1371/journal.pone.0283845 37011101 PMC10069780

[31] Kianersi F, Naderi Beni A, Naderi Beni Z. Clinical manifestation and prognosis of active ocular toxoplasmosis in Iran. Int Ophthalmol. 2012;32:539–545. doi: 10.1007/s10792-012-9599-0 22733253

[32] Opsteegh M, Kortbeek TM, Havelaar AH, van der Giessen JW. Intervention Strategies to Reduce Human *Toxoplasma gondii* Disease Burden. Clin Infect Dis. 2015;101–107. doi: 10.1093/cid/ciu721 25225234

